# Synthesis, crystal structure and Hirshfeld surface analysis of a 1D coordination polymer *catena*-poly[[di­aqua­bis­(nicotinamide-κ*N* ^1^)nickel(II)]-μ-fumarato-κ^2^ *O* ^1^:*O* ^4^]

**DOI:** 10.1107/S2056989018011489

**Published:** 2018-08-16

**Authors:** Sevgi Kansiz, Necmi Dege, Valentina A. Kalibabchuk

**Affiliations:** a Ondokuz Mayıs University, Faculty of Arts and Sciences, Department of Physics, 55139, Kurupelit, Samsun, Turkey; b Department of General Chemistry, O. O. Bohomolets National Medical University, Shevchenko Blvd. 13, 01601 Kiev, Ukraine

**Keywords:** crystal structure, fumaric acid, nicotinamide, nickel(II), Hirshfeld surfaces

## Abstract

The reaction of NiCl_2_ with fumaric acid and nicotinamide in basic solution produces the title polymeric complex, [Ni(C_4_H_2_O_4_)(C_6_H_6_N_2_O)_2_(H_2_O)_2_]_*n*_. The Ni^II^ cation, located on an inversion centre, is coordinated by two O atoms of the fumarate dianions, two N atoms from nicotinamide ligands and two water mol­ecules in a distorted octa­hedral fashion. In the crystal, the fumarate dianions bridge the Ni^II^ cations, forming polymeric chains propagating along the [101] direction; the polymeric chains are further linked by O—H⋯O, N—H⋯O and C—H⋯O hydrogen bonds, forming a three-dimensional supra­molecular architecture. Hirshfeld surface analyses and two-dimensional fingerprint plots were used to analyse the inter­molecular inter­actions present in the crystal, indicating that the most important contributions for the crystal packing are from H⋯O/O⋯H (35.9%), H⋯H (31.7%) and C⋯C (10.4%) inter­actions.

## Abstract 2

In the crystal, the fumarate dianions bridge the Ni^II^ cations into polymeric chains propagating along the [101] direction; these polymeric chains are further linked by O—H⋯O, N—H⋯O and C—H⋯O hydrogen bonds, forming a three-dimensional supra­molecular architecture.

## Chemical context   

Metal complexes of biologically important ligands are sometimes more effective than the free ligands. Many transition and heavy metal cations play an important role in biological processes in the formation of many vitamins and drug components. An important element for biological systems is nickel. Nickel complexes have biological applications such as anti­epileptic, anti­microbial, anti­bacterial and anti­cancer activities (Bombicz *et al.*, 2001 ▸). The metal-ion geometries of coordination compounds can be easily identified. Di­carb­oxy­lic acid ligands are utilized in the synthesis of a range of metal complexes and fumaric acid and amide have been particularly useful in creating many supra­molecular structures (Pavlishchuk *et al.*, 2011 ▸; Ostrowska *et al.*, 2016 ▸), in particular between nicotinamide and a variety of carb­oxy­lic acid mol­ecules. We have prepared a new Ni^II^ complex, *catena*-poly[[di­aqua­bis(nicotinamide-κ*N* ^1^)nickel(II)]-μ-fumarato-κ^2^ *O* ^1^:*O* ^4^], whose structure has been determined by single crystal X-ray diffraction analysis. In addition, to understand the inter­molecular inter­actions in the crystal structure, Hirshfeld surface analysis was performed.

## Structural commentary   

**Figure 1:**
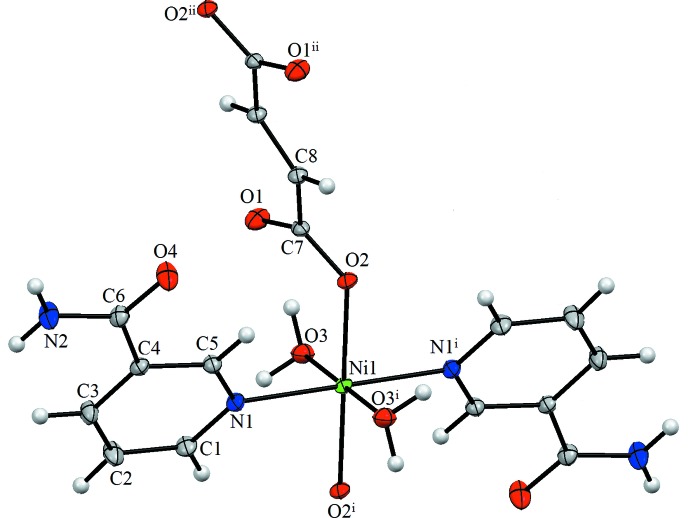
The mol­ecular structure of the asymmetric unit of the title compound, showing the atom labelling. Displacement ellipsoids are drawn at the 20% probability level. [Symmetry codes: (i) −*x* + 1, −*y* + 2, −*z* + 2; (ii) −*x*, −*y* + 1, −*z* + 2.]

The mol­ecular structure of the asymmetric unit of the title compound is illustrated in Fig. 1 ▸. This linear one-dimensional coordination polymer consists of a nickel centre coordinated in an octa­hedral fashion by two oxygen atoms of fumaric acid dianions, two nicotinamide nitro­gen atoms and two aqua ligands.
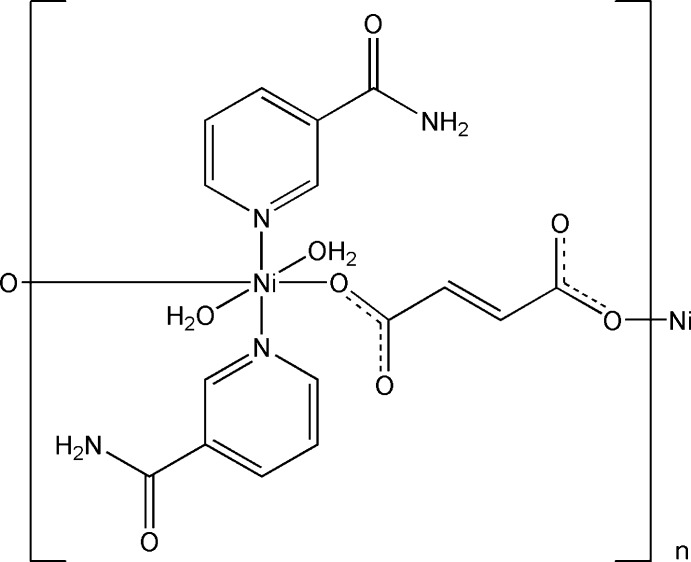


The Ni1—O2, Ni1—O3 and Ni1—N1 bond lengths are 2.0484 (12), 2.0792 (13) and 2.1187 (14) Å, respectively. The C—O bond lengths in the deprotonated carb­oxy­lic groups differ noticeably [C7—O1 = 1.248 (2) Å and C7—O2 = 1.266 (2) Å], which is typical for monodentately coordinated carboxyl­ates (Gumienna-Kontecka *et al.*, 2007 ▸; Pavlishchuk *et al.*, 2010 ▸; Penkova *et al.*, 2010 ▸). In the same way, the C6—O4 bond in the amide group [1.236 (2) Å] shows partial double-bond character. The values of the Ni—O_water_ and Ni—N_pyridine_ bond lengths and the bond angles involving the Ni1 atom (see supporting information) are close to those reported for similar nickel(II) complexes (Krämer *et al.*, 2002 ▸; Bora & Das, 2011 ▸; Moroz *et al.*, 2012 ▸). The conformation of the title compound is best defined by the torsion angles C4—C5—N1—Ni1, O1—C7—O2—Ni1 and C8—C7—O2—Ni1 of 172.22 (13)°, −26.7 (2)° and 151.80 (11)°, respectively.

## Supra­molecular features   

**Figure 2:**
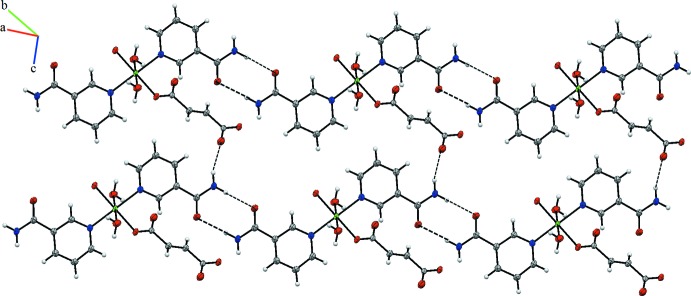
A partial view of the crystal packing of the title compound. Dashed lines indicate the hydrogen bonds (see Table 1 ▸).

In the crystal, the polymeric chains are linked by O—H⋯O, N—H⋯O and C—H⋯O hydrogen bonds (Table 1 ▸), forming a three-dimensional supra­molecular architecture (Fig. 2 ▸). The shortest non-hydrogen-bonding inter­molecular distances of the title compound are 2.870 (2) Å [for O3⋯O4(−*x* + 1, −*y* + 1, −z + 2)] and 2.855 (2) Å [for O3⋯O1(*x* + 1, *y*, *z*)]. The strongest hydrogen-bonded inter­molecular distance is 2.06 Å [H3*A*⋯O4(−*x* + 1, −*y* + 1, −*z* + 2)].

## Hirshfeld surface analysis   

**Figure 3:**
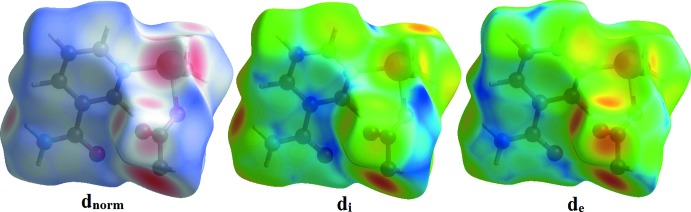
The Hirshfeld surface of the title compound mapped over *d_norm_*, *d*_i_ and *d*_e_.

The Hirshfield surface analysis was performed using the *CrystalExplorer* program (Turner *et al.*, 2017 ▸). The Hirshfeld surfaces and their associated two-dimensional fingerprint plots were used to qu­antify the various inter­molecular inter­actions in the synthesized complex. The Hirshfeld surfaces mapped over *d*_norm_, *d*_i_ and *d*_e_ are shown in Fig. 3 ▸. The red spots indicate the inter­molecular contacts associated with strong hydrogen bonds and inter­atomic contacts (Gümüş *et al.*, 2018 ▸; Kansız & Dege, 2018 ▸; Sen *et al.*, 2018 ▸). For the title compound, these correspond to the near-type H⋯O contacts resulting from O—H⋯O and N—H⋯O hydrogen bonds (Figs. 3 ▸ and 4 ▸). The Hirshfeld surfaces were generated using a standard (high) surface resolution with the three-dimensional *d*_norm_ surface mapped over a fixed colour scale of −1.219 (red) to 1.466 (blue) a.u.

**Figure 5:**
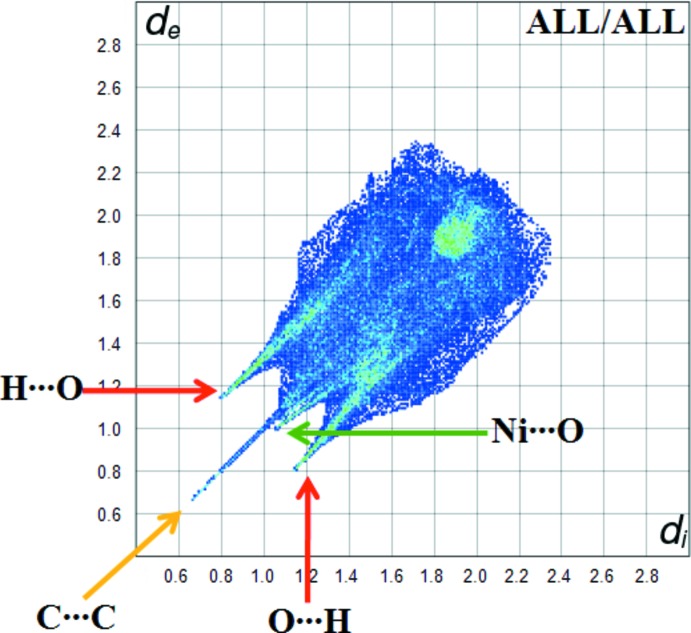
A fingerprint plot of the title compound.

**Figure 6:**
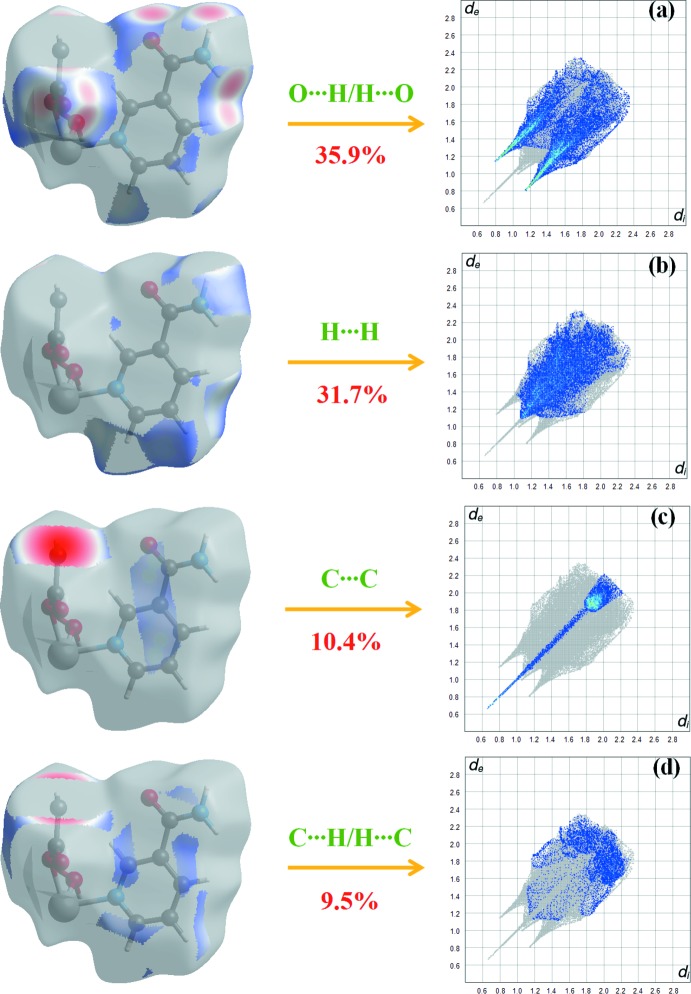
Two-dimensional fingerprint plots with a *d*_norm_ view of the O⋯H/H⋯O (35.9%), H⋯H (31.7%), C⋯C (10.4%) and C⋯H/H⋯C (9.5%) contacts in the title compound.

Fig. 5 ▸ shows the two-dimensional fingerprint of the sum of the contacts contributing to the Hirshfeld surface represented in normal mode. The graph shown in Fig. 6 ▸ *a* represents the O⋯H/H⋯O contacts (35.9%) between the oxygen atoms inside the surface and the hydrogen atoms outside the surface, *d*_e_ + *d*_i_ = 1.9 Å, and two symmetrical points at the top, bottom left and right. These are characteristic of O—H⋯O and N—H⋯O hydrogen bonds. Fig. 6 ▸ *b* (H⋯H) shows the two-dimensional fingerprint of the (*d*_i_, *d*_e_) points associated with hydrogen atoms. It is characterized by an end point that points to the origin and corresponds to *d*_i_ = *d*_e_ = 1.08 Å, which indicates the presence of the H⋯H contacts in this study (31.7%). The graph shown in Fig. 6 ▸ *d* (C⋯H/H⋯C) shows the contact between the carbon atoms inside the surface and the hydrogen atoms outside the Hirshfeld surface and *vice versa* (9.5%). In addition, C⋯C (10.4%), N⋯H/H⋯N (3.6%) and Ni⋯O/O⋯Ni (3.4%) contacts contribue to the Hirshfeld surface.

## Synthesis and crystallization   

A solution of NaOH (52 mmol, 2.07 g) was added to an aqueous solution of H_2_Fum (26 mmol, 3 g) with stirring. A solution of NiCl_2_·6H_2_O (26 mmol, 6.14 g) in ethanol was added. The mixture was heated at 353 K for an hour and then the pink mixture was filtered and left to dry at room temperature. The reaction mixture (0.88 mmol, 0.20 g) was dissolved in ethanol and added to a methanol solution of nicotinamide (1.76 mmol, 0.21 g). The mixture was heated at 353 K for 30 min with stirring and the resulting suspension was filtered. On slow evaporation of the filtrate, over a period of three weeks, blue block-shaped crystals of the title complex were obtained.

## Refinement   

Crystal data, data collection and structure refinement details are summarized in Table 2 ▸. The C-bound H atoms were positioned geometrically and refined using a riding model: C—H = 0.93 Å with *U*_iso_(H) = 1.2*U*_eq_(C).

## Supplementary Material

Crystal structure: contains datablock(s) I, global. DOI: 10.1107/S2056989018011489/xu5933sup1.cif

Structure factors: contains datablock(s) I. DOI: 10.1107/S2056989018011489/xu5933Isup2.hkl

CCDC reference: 1563283

Additional supporting information:  crystallographic information; 3D view; checkCIF report

## Figures and Tables

**Figure 4:**
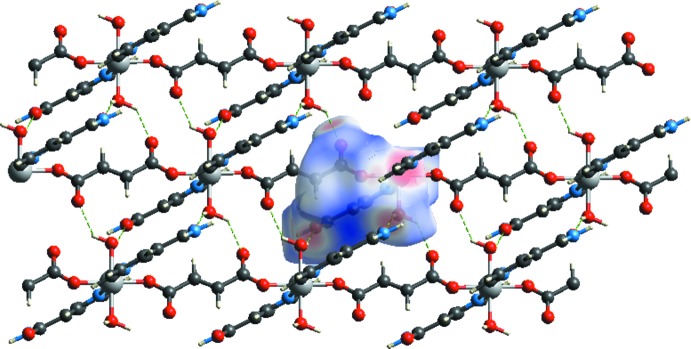
Hirshfeld surfaces mapped over *d_norm_* to visualize the inter­molecular inter­actions of the title compound.

**Table 1:** Hydrogen-bond geometry (Å, °)

*D*—H⋯*A*	*D*—H	H⋯*A*	*D*⋯*A*	*D*—H⋯*A*
O3—H3*A*⋯O4^ii^	0.85	2.06	2.8699 (19)	159
O3—H3*B*⋯O1^iii^	0.85	2.17	2.8550 (19)	138
O3—H3*B*⋯O1^i^	0.85	2.30	2.9916 (18)	138
N2—H2*A*⋯O4^iv^	0.86	2.16	2.980 (2)	158
N2—H2*B*⋯O1^v^	0.86	2.10	2.929 (2)	161
C3—H3⋯O1^v^	0.93	2.40	3.296 (2)	162

Symmetry codes: (i) 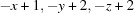; (ii) 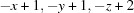; (iii) 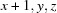; (iv) 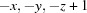; (v) 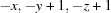.

**Table 2:** Experimental details

Crystal data	
Chemical formula	[Ni(C_4_H_2_O_4_)(C_6_H_6_N_2_O)_2_(H_2_O)_2_]
*M*_r_	453.05
Crystal system, space group	Triclinic, *P* 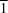
Temperature (K)	296
*a*, *b*, *c* (Å)	7.3660 (5), 7.5521 (5), 8.9344 (6)
α, β, γ (°)	109.672 (2), 102.556 (2), 98.887 (2)
*V* (Å^3^)	442.57 (5)
*Z*	1
Radiation type	Mo *K*α
μ (mm^−1^)	1.15
Crystal size (mm)	0.21 × 0.17 × 0.14

Data collection	
Diffractometer	Stoe *IPDS* 2
Absorption correction	Analytical (*X-RED32*; Stoe & Cie, 2002 ▸)
*T*_min_, *T*_max_	0.622, 0.746
No. of measured, independent and observed [*I* > 2σ(*I*)] reflections	20590, 2203, 2075
*R*_int_	0.029
(sin θ/λ)_max_ (Å^−1^)	0.669

Refinement	
*R*[*F* ^2^ > 2σ(*F* ^2^)], *wR*(*F* ^2^), *S*	0.029, 0.076, 1.09
No. of reflections	2203
No. of parameters	134
H-atom treatment	H-atom parameters constrained
Δρ_max_, Δρ_min_ (e Å^−3^)	0.33, −0.51

Computer programs: *X-AREA* (Stoe & Cie, 2002 ▸), *SHELXT2014* (Sheldrick, 2015*a* ▸), *SHELXL2016* (Sheldrick, 2015*b* ▸), *ORTEP-3 for Windows* and *WinGX* (Farrugia, 2012 ▸) and *PLATON* (Spek, 2009 ▸).

## supplementary crystallographic information

### Crystal data

**Table:**

[Ni(C_4_H_2_O_4_)(C_6_H_6_N_2_O)_2_(H_2_O)_2_]	*Z* = 1
*M**_r_* = 453.05	*F*(000) = 234
Triclinic, *P*1	*D*_x_ = 1.700 Mg m^−^^3^
*a* = 7.3660 (5) Å	Mo *K*α radiation, λ = 0.71073 Å
*b* = 7.5521 (5) Å	Cell parameters from 9881 reflections
*c* = 8.9344 (6) Å	θ = 3.0–28.3°
α = 109.672 (2)°	µ = 1.15 mm^−^^1^
β = 102.556 (2)°	*T* = 296 K
γ = 98.887 (2)°	Block, blue
*V* = 442.57 (5) Å^3^	0.21 × 0.17 × 0.14 mm

### Data collection

**Table:**

Stoe IPDS 2 diffractometer	2075 reflections with *I* > 2σ(*I*)
φ and ω scans	*R*_int_ = 0.029
Absorption correction: analytical (X-RED32; Stoe & Cie, 2002)	θ_max_ = 28.4°, θ_min_ = 2.9°
*T*_min_ = 0.622, *T*_max_ = 0.746	*h* = −9→9
20590 measured reflections	*k* = −10→10
2203 independent reflections	*l* = −11→11

### Refinement

**Table:**

Refinement on *F*^2^	0 restraints
Least-squares matrix: full	Hydrogen site location: mixed
*R*[*F*^2^ > 2σ(*F*^2^)] = 0.029	H-atom parameters constrained
*wR*(*F*^2^) = 0.076	*w* = 1/[σ^2^(*F*_o_^2^) + (0.0376*P*)^2^ + 0.3311*P*] where *P* = (*F*_o_^2^ + 2*F*_c_^2^)/3
*S* = 1.09	(Δ/σ)_max_ < 0.001
2203 reflections	Δρ_max_ = 0.33 e Å^−^^3^
134 parameters	Δρ_min_ = −0.51 e Å^−^^3^

### Special details

**Table:**

Geometry. All esds (except the esd in the dihedral angle between two l.s. planes) are estimated using the full covariance matrix. The cell esds are taken into account individually in the estimation of esds in distances, angles and torsion angles; correlations between esds in cell parameters are only used when they are defined by crystal symmetry. An approximate (isotropic) treatment of cell esds is used for estimating esds involving l.s. planes.

### Fractional atomic coordinates and isotropic or equivalent isotropic displacement parameters (Å^2^)

**Table:**

	*x*	*y*	*z*	*U*_iso_*/*U*_eq_	
Ni1	0.500000	1.000000	1.000000	0.01693 (10)	
O2	0.33073 (17)	0.81811 (18)	1.06897 (15)	0.0230 (3)	
O1	0.03134 (19)	0.80242 (19)	0.93228 (17)	0.0288 (3)	
O3	0.69940 (18)	0.84277 (19)	1.04390 (16)	0.0265 (3)	
H3A	0.714414	0.842222	1.140797	0.040*	
H3B	0.805970	0.895352	1.036435	0.040*	
O4	0.1845 (2)	0.2218 (2)	0.65461 (17)	0.0365 (3)	
N1	0.4051 (2)	0.7960 (2)	0.75121 (17)	0.0208 (3)	
N2	0.0821 (3)	0.1382 (2)	0.3790 (2)	0.0344 (4)	
H2A	0.030092	0.020641	0.362653	0.041*	
H2B	0.075841	0.173000	0.296112	0.041*	
C8	0.0907 (2)	0.5495 (2)	1.0232 (2)	0.0201 (3)	
H8	0.185109	0.498535	1.069060	0.024*	
C7	0.1527 (2)	0.7390 (2)	1.00643 (19)	0.0179 (3)	
C4	0.2550 (2)	0.4705 (2)	0.5543 (2)	0.0201 (3)	
C5	0.3392 (2)	0.6063 (2)	0.7149 (2)	0.0205 (3)	
H5	0.350571	0.563601	0.801926	0.025*	
C3	0.2450 (3)	0.5333 (3)	0.4242 (2)	0.0280 (4)	
H3	0.190437	0.446164	0.314732	0.034*	
C6	0.1722 (3)	0.2659 (2)	0.5321 (2)	0.0228 (3)	
C1	0.3942 (3)	0.8543 (3)	0.6243 (2)	0.0258 (4)	
H1	0.440043	0.985427	0.647565	0.031*	
C2	0.3180 (3)	0.7284 (3)	0.4605 (2)	0.0317 (4)	
H2	0.315654	0.773682	0.375558	0.038*	

### Atomic displacement parameters (Å^2^)

**Table:**

	*U*^11^	*U*^22^	*U*^33^	*U*^12^	*U*^13^	*U*^23^
Ni1	0.01813 (15)	0.01127 (14)	0.01788 (16)	−0.00304 (10)	0.00371 (11)	0.00516 (11)
O2	0.0213 (6)	0.0206 (6)	0.0234 (6)	−0.0051 (5)	0.0041 (5)	0.0100 (5)
O1	0.0261 (6)	0.0247 (6)	0.0374 (7)	0.0034 (5)	0.0054 (5)	0.0176 (6)
O3	0.0237 (6)	0.0306 (7)	0.0305 (7)	0.0067 (5)	0.0098 (5)	0.0168 (6)
O4	0.0547 (9)	0.0216 (6)	0.0254 (7)	−0.0050 (6)	0.0003 (6)	0.0128 (5)
N1	0.0232 (7)	0.0164 (6)	0.0187 (6)	−0.0006 (5)	0.0042 (5)	0.0053 (5)
N2	0.0527 (11)	0.0158 (7)	0.0227 (8)	−0.0083 (7)	0.0013 (7)	0.0061 (6)
C8	0.0212 (8)	0.0164 (7)	0.0216 (8)	−0.0002 (6)	0.0054 (6)	0.0087 (6)
C7	0.0207 (7)	0.0133 (7)	0.0174 (7)	−0.0012 (6)	0.0067 (6)	0.0046 (6)
C4	0.0210 (8)	0.0157 (7)	0.0212 (8)	0.0005 (6)	0.0050 (6)	0.0065 (6)
C5	0.0242 (8)	0.0168 (7)	0.0190 (7)	0.0013 (6)	0.0053 (6)	0.0071 (6)
C3	0.0363 (10)	0.0205 (8)	0.0187 (8)	−0.0043 (7)	0.0021 (7)	0.0057 (7)
C6	0.0269 (8)	0.0157 (7)	0.0226 (8)	0.0013 (6)	0.0037 (6)	0.0073 (6)
C1	0.0309 (9)	0.0168 (8)	0.0252 (9)	−0.0037 (7)	0.0050 (7)	0.0086 (7)
C2	0.0454 (11)	0.0240 (9)	0.0218 (9)	−0.0038 (8)	0.0045 (8)	0.0125 (7)

### Geometric parameters (Å, º)

**Table:**

Ni1—O2^i^	2.0484 (12)	N2—H2A	0.8600
Ni1—O2	2.0484 (12)	N2—H2B	0.8600
Ni1—O3	2.0792 (13)	C8—C8^ii^	1.326 (3)
Ni1—O3^i^	2.0792 (13)	C8—C7	1.500 (2)
Ni1—N1	2.1187 (14)	C8—H8	0.9300
Ni1—N1^i^	2.1187 (14)	C4—C5	1.386 (2)
O2—C7	1.266 (2)	C4—C3	1.388 (2)
O1—C7	1.248 (2)	C4—C6	1.500 (2)
O3—H3A	0.8501	C5—H5	0.9300
O3—H3B	0.8501	C3—C2	1.387 (3)
O4—C6	1.236 (2)	C3—H3	0.9300
N1—C1	1.340 (2)	C1—C2	1.379 (3)
N1—C5	1.341 (2)	C1—H1	0.9300
N2—C6	1.327 (2)	C2—H2	0.9300
			
O2^i^—Ni1—O2	180.0	C8^ii^—C8—C7	124.0 (2)
O2^i^—Ni1—O3	96.22 (5)	C8^ii^—C8—H8	118.0
O2—Ni1—O3	83.78 (5)	C7—C8—H8	118.0
O2^i^—Ni1—O3^i^	83.78 (5)	O1—C7—O2	126.09 (15)
O2—Ni1—O3^i^	96.22 (5)	O1—C7—C8	119.43 (14)
O3—Ni1—O3^i^	180.0	O2—C7—C8	114.47 (14)
O2^i^—Ni1—N1	89.41 (5)	C5—C4—C3	118.21 (15)
O2—Ni1—N1	90.59 (5)	C5—C4—C6	117.84 (15)
O3—Ni1—N1	86.92 (5)	C3—C4—C6	123.89 (15)
O3^i^—Ni1—N1	93.08 (5)	N1—C5—C4	123.40 (15)
O2^i^—Ni1—N1^i^	90.59 (5)	N1—C5—H5	118.3
O2—Ni1—N1^i^	89.41 (5)	C4—C5—H5	118.3
O3—Ni1—N1^i^	93.08 (5)	C2—C3—C4	118.71 (16)
O3^i^—Ni1—N1^i^	86.92 (5)	C2—C3—H3	120.6
N1—Ni1—N1^i^	180.00 (8)	C4—C3—H3	120.6
C7—O2—Ni1	128.31 (11)	O4—C6—N2	122.05 (16)
Ni1—O3—H3A	109.5	O4—C6—C4	120.06 (16)
Ni1—O3—H3B	109.3	N2—C6—C4	117.87 (15)
H3A—O3—H3B	109.5	N1—C1—C2	122.83 (16)
C1—N1—C5	117.63 (15)	N1—C1—H1	118.6
C1—N1—Ni1	120.90 (11)	C2—C1—H1	118.6
C5—N1—Ni1	121.25 (11)	C1—C2—C3	119.17 (17)
C6—N2—H2A	120.0	C1—C2—H2	120.4
C6—N2—H2B	120.0	C3—C2—H2	120.4
H2A—N2—H2B	120.0		
			
Ni1—O2—C7—O1	−26.7 (2)	C6—C4—C3—C2	176.66 (18)
Ni1—O2—C7—C8	151.80 (11)	C5—C4—C6—O4	−1.8 (3)
C8^ii^—C8—C7—O1	−6.3 (3)	C3—C4—C6—O4	−178.95 (19)
C8^ii^—C8—C7—O2	175.1 (2)	C5—C4—C6—N2	176.32 (17)
C1—N1—C5—C4	−2.4 (3)	C3—C4—C6—N2	−0.8 (3)
Ni1—N1—C5—C4	172.22 (13)	C5—N1—C1—C2	0.3 (3)
C3—C4—C5—N1	2.5 (3)	Ni1—N1—C1—C2	−174.31 (16)
C6—C4—C5—N1	−174.83 (15)	N1—C1—C2—C3	1.5 (3)
C5—C4—C3—C2	−0.5 (3)	C4—C3—C2—C1	−1.4 (3)

Symmetry codes: (i) −*x*+1, −*y*+2, −*z*+2; (ii) −*x*, −*y*+1, −*z*+2.

### Hydrogen-bond geometry (Å, º)

**Table:**

*D*—H···*A*	*D*—H	H···*A*	*D*···*A*	*D*—H···*A*
O3—H3*A*···O4^iii^	0.85	2.06	2.8699 (19)	159
O3—H3*B*···O1^iv^	0.85	2.17	2.8550 (19)	138
O3—H3*B*···O1^i^	0.85	2.30	2.9916 (18)	138
N2—H2*A*···O4^v^	0.86	2.16	2.980 (2)	158
N2—H2*B*···O1^vi^	0.86	2.10	2.929 (2)	161
C3—H3···O1^vi^	0.93	2.40	3.296 (2)	162

Symmetry codes: (i) −*x*+1, −*y*+2, −*z*+2; (iii) −*x*+1, −*y*+1, −*z*+2; (iv) *x*+1, *y*, *z*; (v) −*x*, −*y*, −*z*+1; (vi) −*x*, −*y*+1, −*z*+1.
